# Real-world incidence of G3-G4 adverse events in patients with advanced renal cell carcinoma receiving immune-combinations (ARON-1)

**DOI:** 10.3389/fimmu.2026.1805104

**Published:** 2026-04-20

**Authors:** Evelyn L. Beas-Lozano, Sarah Scagliarini, Paola I. Valdez-Sandoval, María Fernanda Esparza-Orozco, Mehmet Asin Bilen, Aristotelis Bamias, Haoran Li, Maria José Juan Fita, Jindrich Kopecky, Ray Manneh Kopp, Marwan Ghosn, Andre Poisl Fay, Dipen Bhuva, Jakub Kucharz, Thomas Büttner, Javier Molina-Cerrillo, Ondrej Fiala, Alessandro Rizzo, Brigida Anna Maiorano, Andrey Soares, Sebastiano Buti, Fernando Sabino Marques Monteiro, Francesco Massari, Matteo Santoni, Maria T. Bourlon

**Affiliations:** 1 Department of Hematology and Oncology, Instituto Nacional de Ciencias Médicas y Nutrición Salvador Zubirán, Mexico City, Mexico; 2 Department of Medical Oncology, AORN “A. Cardarelli”, Naples, Italy; 3 Department of Hematology and Oncology, Emory University School of Medicine, Atlanta, GA, United States; 4 2^nd^ Propaedeutic Department of Internal Medicine, ATTIKON University Hospital, School of Medicine, National and Kapodistrian University of Athens, Athens, Greece; 5 Division of Medical Oncology, Department of Internal Medicine, University of Kansas Cancer Center, Kansas City, MO, United States; 6 Fundación Instituto Valenciano de Oncología, Profesor Beltrán Baguena, Valencia, Spain; 7 Department of Clinical Oncology and Radiotherapy, University Hospital Hradec Kralove, Hradec Kralove, Czechia; 8 Department of Clinical Oncology, Clinical Oncology, Sociedad de Oncología y Hematología del Cesar, Valledupar, Colombia; 9 Hematology-Oncology Department, Faculty of Medicine, Saint Joseph University of Beirut, Beirut, Lebanon; 10 Pontifícia Universidade Católica do Rio Grande do Sul (PUCRS) School of Medicine, Porto Alegre, RS, Brazil; 11 Department of Medical Oncology, Army Hospital Research and Referral, New Delhi, India; 12 Department of Uro-Oncology, Maria Sklodowska-Curie National Research Institute of Oncology Warsaw, Warsaw, Poland; 13 Department of Urology, University Hospital Bonn (UKB), Bonn, Germany; 14 Department of Medical Oncology, Hospital Ramón y Cajal, Madrid, Spain; 15 Department of Oncology and Radiotherapeutics, Faculty of Medicine and University Hospital in Pilsen, Charles University, Pilsen, Czechia; 16 Biomedical Center, Faculty of Medicine in Pilsen, Charles University, Pilsen, Czechia; 17 S.S.D. C.O.r.O. Bed Management Presa in Carico, TDM, Istituto di Ricovero e Cura a Carattere Scientifico (IRCCS) Istituto Tumori “Giovanni Paolo II”, Bari, Italy; 18 Department of Medical Oncology, Istituto di Ricovero e Cura a Carattere Scientifico (IRCCS) San Raffaele Hospital, Milan, Italy; 19 Oncology Unit, Eistein Hospital Israelita, São Paulo, SP, Brazil; 20 Latin American Cooperative Oncology Group - LACOG, Porto Alegre, Brazil; 21 Medical Oncology Unit, University Hospital of Parma, Parma, Italy; 22 Department of Medicine and Surgery, University of Parma, Parma, Italy; 23 Oncology and Hematology Department, Hospital Sírio Libanês, Brasília, Brazil; 24 Medical Oncology, Istituto di Ricovero e Cura a Carattere Scientifico (IRCCS) Azienda Ospedaliero-Universitaria di Bologna, Bologna, Italy; 25 Department of Medical and Surgical Sciences (DIMEC), University of Bologna, Bologna, Italy; 26 Medical Oncology Unit, Macerata Hospital, Macerata, Italy

**Keywords:** renal cell carcinoma, immune checkpoint inhibitors, real-world evidence, treatment-related adverse events, tyrosine kinase inhibitors

## Abstract

**Background:**

Immune-based combination therapies have become the standard first-line treatment for metastatic renal cell carcinoma (mRCC) and have positively impacted survival outcomes in phase III clinical trials. However, these trials are conducted in highly selected populations and controlled settings, which may limit the generalizability of toxicity profiles to routine clinical practice. Real-world data are therefore essential to better characterize the incidence and determinants of severe adverse events (AEs) associated with immune-based combinations.

**Methods:**

We conducted a multinational, retrospective analysis of the ARON-1 registry, of patients with mRCC who received first-line immune-based combination therapy across 17 countries. The primary endpoint was to evaluate the real-world incidence of grade 3–4 (G3-G4) AEs. Logistic regression analyses were performed to identify clinical factors associated with toxicity. Overall survival (OS) was assessed using Kaplan–Meier methods, with landmark analyses to explore the association between G3-G4 AEs and survival outcomes.

**Results:**

Among 2, 401 patients receiving immune-based combinations, 1, 921 (80%) had complete data on grade 3–4 AEs and were included in the analysis. G3–G4 AEs occurred in 34% (n=653). Pembrolizumab plus lenvatinib was associated with the highest incidence of high-grade AEs, whereas nivolumab plus ipilimumab showed the lowest. Older age and female sex were independently associated with an increased risk of G3–G4 toxicity. Although the occurrence of severe AEs was associated with improved OS in unadjusted analyses, this association was non-significant in the 6-month landmark analyses.

**Conclusion:**

In this large, multinational real-world cohort, the incidence of G3–G4 adverse events in patients with mRCC treated with immune-based combinations was lower than that reported in pivotal clinical trials, underscoring meaningful differences between trial and routine practice settings. Patient- and regimen-specific factors significantly influenced toxicity risk. These findings highlight the complementary role of real-world evidence in informing toxicity management and support individualized treatment strategies to optimize outcomes in everyday clinical practice.

## Introduction

The global incidence of kidney cancer was estimated to increase by approximately 29, 000 cases compared with 2022, accounting for nearly 166, 000 deaths worldwide, an increase of about 10, 000 deaths relative to 2022 (1). Moreover, renal cell carcinoma (RCC) cases are projected to increase by 72% in 2050, with an estimated 304, 861 new deaths worldwide, largely driven by population growth and aging; however, this projection may also be influenced by additional factors, including modifiable and non-modifiable risk factors, as well as treatment access (2). Following the advent of first-line immune-based combinations for metastatic renal cell carcinoma (mRCC), the risk of death has decreased by 26% (HR0.74, 95%CI0.67e0.81, P<0.001) compared with the previous standard of care, sunitinib (3). Currently, four immune-based combinations are commonly used as first-line treatment of mRCC patients: nivolumab plus ipilimumab, pembrolizumab plus axitinib, pembrolizumab plus lenvatinib, and nivolumab plus cabozantinib. In pivotal clinical trials, these regimens have demonstrated median overall survival (OS) ranging from 46.5 to 53.7 months (4–7). However, real-world outcomes may differ, showing an estimated median OS of 36.5 months (95% CI 24.8−60.8) in a previous ARON-1 report (8). Similarly, immune based combinations have shown improvement in outcomes for non–clear cell RCC, which include nivolumab plus ipilimumab, pembrolizumab plus lenvatinib, and nivolumab plus cabozantinib (9–12).

Although these combinations are associated with maintenance or improvement in health-related quality of life (HrQL) compared to sunitinib monotherapy, treatment-related adverse events (AEs) remain common and treatment-related mortality is not negligible (13–16). AEs occur in approximately 93–99% of patients; however, grade ≥3 events are reported in 46-82% across pivotal trials (5–7). Importantly, each regimen exhibits a distinct toxicity profile. While the spectrum of immune-related adverse events (irAEs) is largely comparable across immunotherapy-tyrosine kinase inhibitor (IO/TKI) regimens, the nivolumab plus ipilimumab combination is associated with higher rates of irAEs, particularly during the initial treatment phase, leading to treatment discontinuation in 22% of patients and the use of high-dose glucocorticoids (≥40 mg of prednisone) in 35% of patients (5). While typical TKI–related adverse events include cutaneous, gastrointestinal, cardiovascular, hepatic, endocrine, renal, and pulmonary events, their frequency and severity vary according to the specific agent used (17). For instance, in the KEYNOTE-426, grade ≥3 hypertension occurred in 22% of patients, likely attributed to axitinib, whereas, in CheckMate 9ER, hand–foot syndrome was observed in 7.5% of patients receiving cabozantinib-based therapy. Dose reductions of TKIs were common across phase III clinical trials; axitinib demonstrated more favorable tolerability, with 20% of patients requiring dose reductions, whereas cabozantinib and lenvatinib required dose reductions in 56% and 68% of patients, respectively (4–7).

However, overlapping toxicities pose a significant challenge for cancer care providers. In the CLEAR trial, the most frequently reported AEs were diarrhea (61.4%), hypertension (55%), and hypothyroidism (47%) (7). While hypertension is likely attributable to lenvatinib, diarrhea and hypothyroidism require a comprehensive multidisciplinary clinical assessment to distinguish between irAEs and TKI-associated toxicities. This assessment is particularly important to determine appropriate treatment interruptions and optimizing therapy resumption to preserve quality of life and maximize overall survival.

Although phase III clinical trials were the basis for the approval of these immune-based combinations for mRCC, their findings are derived from highly selected patient populations treated in controlled environments. In contrast, real-world data capture the heterogeneity of routine clinical practice, where patient characteristics, comorbidities, treatment delivery, and the identification and management of AEs may differ substantially from trial settings. In this context, multinational real-world evidence from the ARON-1 registry, provides pivotal information to characterize the burden of grade 3–4 adverse events associated with the current standard of care for mRCC in everyday clinical practice.

## Patients and methods

### Study population

This retrospective study analyzed adult patients (aged 18 years and older) diagnosed with mRCC, confirmed by histopathological analysis or imaging. The data set included individuals who began first-line immune-based combination treatment with nivolumab plus ipilimumab, pembrolizumab plus axitinib, pembrolizumab plus lenvatinib, or nivolumab plus cabozantinib between January 1, 2021, and April 1, 2025, across 56 centers in 17 different countries. We restricted our analysis to patients receiving first-line nivolumab plus ipilimumab or pembrolizumab plus axitinib or pembrolizumab plus lenvatinib or nivolumab plus cabozantinib. Indeed, in the context of the ARON-1 dataset, only the immune-based combinations that were approved and routinely used in the participating centers during the study period were included in the analysis. Other combinations, such as avelumab plus axitinib and triplet regimens (e.g., ipilimumab plus nivolumab plus cabozantinib), were not included because they were either not approved or not routinely available in the countries contributing to the dataset during the study timeframe.

Patients received first-line therapy until disease progression (either clinically or radiological), unacceptable treatment-related toxicity, or death. Follow-up imaging assessments CT-scans or MRI were performed every 8 to 12 weeks, in line with each institution’s protocol. Routine clinical assessments, including physical exams and laboratory tests, were conducted every 4 to 6 weeks.

Grade 3-4 (G3-G4) AEs were defined according to the Common Terminology Criteria for Adverse Events (CTCAE) v5.0 (18).

Patient information was retrospectively collected from both electronic and paper medical records and included demographic data, tumor histology, the International RCC Database Consortium (IMDC) risk stratification, history of nephrectomy, sites of metastases, G3-G4 treatment-emergent AEs, treatment response, and subsequent therapies. Patients without complete tumor assessment data or those lost to follow-up were excluded from the analysis. Furthermore, patients with missing information on AEs were excluded from the primary analysis, as AEs occurrence represented the main outcome of the study.

### Study endpoints and statistical analysis

This analysis, part of the ARON-1 study, aimed to assess the incidence of G3-G4 among patients undergoing first-line immune-based combination therapies for mRCC. OS was defined as the time from therapy initiation until death from any cause. Time on Treatment (ToT) was defined as the time from therapy initiation until progression or treatment interruption from any cause. Patients who were lost to follow-up were censored at the date of their last documented assessment.

Statistical analysis was conducted using MedCalc software (version 19.6.4; MedCalc Software, Broekstraat 52, 9030 Mariakerke, Belgium). Logistic regression analyses were performed using the entire ARON-1 dataset to evaluate the association between clinical variables and the risk of G3-G4 AEs among patients receiving first-line immune-based combination therapy. Covariates included sex, age, and body mass index (BMI). Odds ratios (ORs) with corresponding 95% confidence intervals were estimated for each variable in both univariate and multivariate models. OS was estimated using Kaplan-Meier survival curves, with 95% CIs calculated via Rothman’s method. The log-rank test was applied for comparing survival outcomes between groups. To minimize bias related to treatment duration, a landmark analysis was conducted with a predefined 6-month time point.

Cox proportional hazards regression models were employed for both univariate and multivariate analyses to explore prognostic variables, with hazard ratios (HRs) and corresponding 95% CIs reported. Logistic regression was used to evaluate the impact of various clinicopathologic factors such as sex, age, and BMI. Categorical variables were analyzed using the chi-square test or Fisher’s exact test where applicable. A two-tailed p-value <0.05 was considered statistically significant.

### Ethical approval

The ARON-1 study protocol received approval from the Ethics Committee of the Marche Region (reference number: 2021-492) as well as from the Institutional Review Boards of all participating centers. The study was carried out in accordance with local laws, Good Clinical Practice (GCP) standards, and international ethical guidelines, including the Declaration of Helsinki principles for research involving human participants.

## Results

### Study cohort

Of the 2, 401 patients from the ARON-1 registry across 17 countries (**Supplementary Figure S1**) who received immune-based combination therapy, 1, 921 individuals (80%) had complete data regarding G3-G4 AEs and were included in the present analysis (**Supplementary Figure S2**). The median age was 63 years (range 20-92). Males represented 74% of the population, whereas females comprised 26%.

Clear cell RCC was the predominant histological subtype, accounting 85% of the cohort. Sarcomatoid differentiation was identified in 15% of patients. Prior nephrectomy had been performed in 1, 194 patients (62%). Lung metastases were the most common, reported in 65% of cases, followed by bone and liver metastases (34% and 19%, respectively). More than half of the patients had *de novo* metastatic disease (56%).

Based on the IMDC risk stratification, 257 patients (13%) were classified as favorable risk disease, 1, 152 (60%) as intermediate risk, and 512 (27%) as poor risk. Nivolumab plus ipilimumab was administered to 781 patients (41%), pembrolizumab plus axitinib 589 patients (31%) and nivolumab plus cabozantinib 336 patients (17%). Pembrolizumab plus lenvatinib was the least frequently used regimen, prescribed to 215 patients (11%). **Table 1** outlines detailed patient characteristics. Among the four treatment regimens, a statistically significant difference was observed only in IMDC risk distribution, with a lower proportion of favorable-risk patients in the nivolumab plus ipilimumab group (**Table 1**).

**Table 1:** Patient characteristics.

Characteristics	Overall no. (%)	Nivolumab / Ipilimumab (%)	Pembrolizumab / Axitinib (%)	Nivolumab / Cabozantinib (%)	Pembrolizumab / Lenvatinib (%)	*p*
**Total patients**	1921 (100)	781 (100)	589 (100)	336 (100)	215 (100)	–
Sex						
Male Female	1415 (74) 506 (26)	569 (73) 212 (27)	449 (76) 140 (24)	245 (73) 91 (27)	152 (71) 63 (29)	0.839
Age (years)						
Median (range)	63 (28–92)	66 (30–88)	65 (34-92)	62 (31-89)	60 (28-85)	–
ECOG-PS						
0-1 ≥2	1794 (93) 127 (7)	721 (92) 60 (8)	559 (95) 30 (5)	315 (94) 21 (6)	199 (93) 16 (7)	0.856
Histologic type						
Clear cell histology Papillary Cromophobe Others	1624 (85) 110 (6) 28 (1) 159 (8)	687 (88) 21 (3) 9 (1) 64 (8)	498 (85) 36 (6) 9 (1) 46 (8)	267 (79) 36 (10) 4 (2) 29 (9)	172 (80) 17 (8) 6 (3) 20 (9)	0.709
Sarcomatoid de-differentiation						
Present	280 (15)	159 (20)	57 (10)	39 (12)	25 (11)	0.141
History of surgery						
Previous nephrectomy	1194 (62)	486 (62)	374 (63)	198 (59)	136 (63)	0.929
Site of metastasis						
Lung Distant lymph nodes Bone Liver Brain	1258 (65) 686 (36) 645 (34) 358 (19) 131 (7)	542 (69) 278 (36) 242 (31) 131 (17) 54 (7)	381 (65) 204 (35) 198 (34) 105 (18) 47 (8)	202 (60) 122 (36) 133 (40) 72 (21) 16 (5)	133 (62) 82 (38) 72 (33) 50 (23) 14 (7)	0.573 0.977 0.572 0.238 0.789
Metastasis at diagnosis						
Yes	1082 (56)	476 (61)	328 (56)	175 (52)	103 (48)	0.054
IMDC Risk Group						
Favorable Intermediate Poor	257 (13) 1152 (60) 512 (27)	22 (3) 531 (68) 228 (29)	124 (21) 344 (58) 121 (21)	51 (15) 175 (52) 110 (33)	60 (28) 102 (47) 53 (25)	**<0.001**

Bold values indicate statistical significance (p < 0.05)

### Incidence of G3-G4 adverse events

The median follow-up was 19.1 months (95%CI 17.9–111.4). The median ToT was 10.5 months (95%CI 5.7–17.6) for nivolumab plus ipilimumab, 11.9 months (95%CI 9.9–13.3) for pembrolizumab plus axitinib, 15.4 months (95%CI 11.0–20.8) for nivolumab plus cabozantinib and 17.3 months (95%CI 13.0–26.2) for pembrolizumab plus lenvatinib.

**Figure 1:**
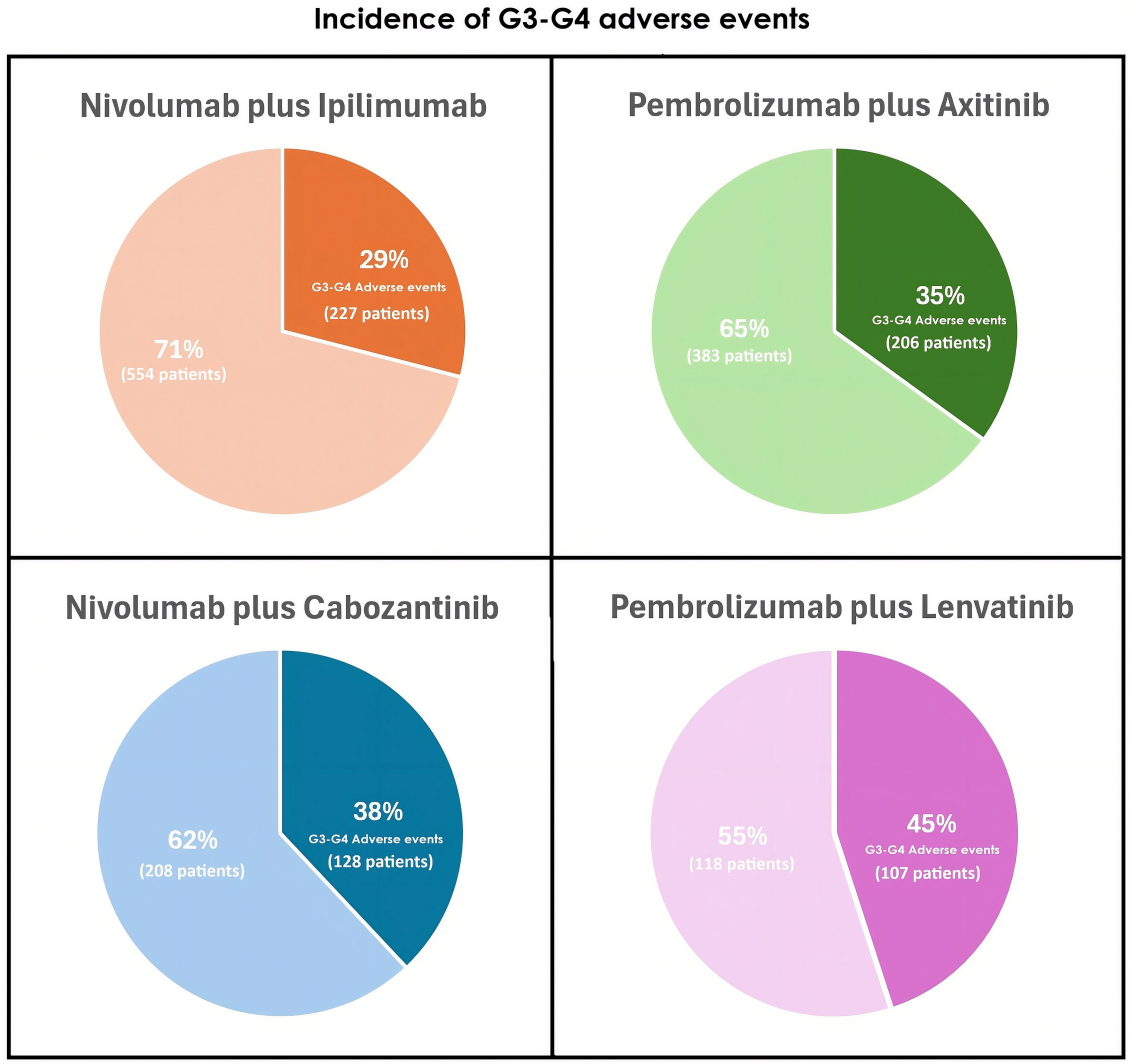
Incidence of G3-G4 adverse events.

**Figure 2:**
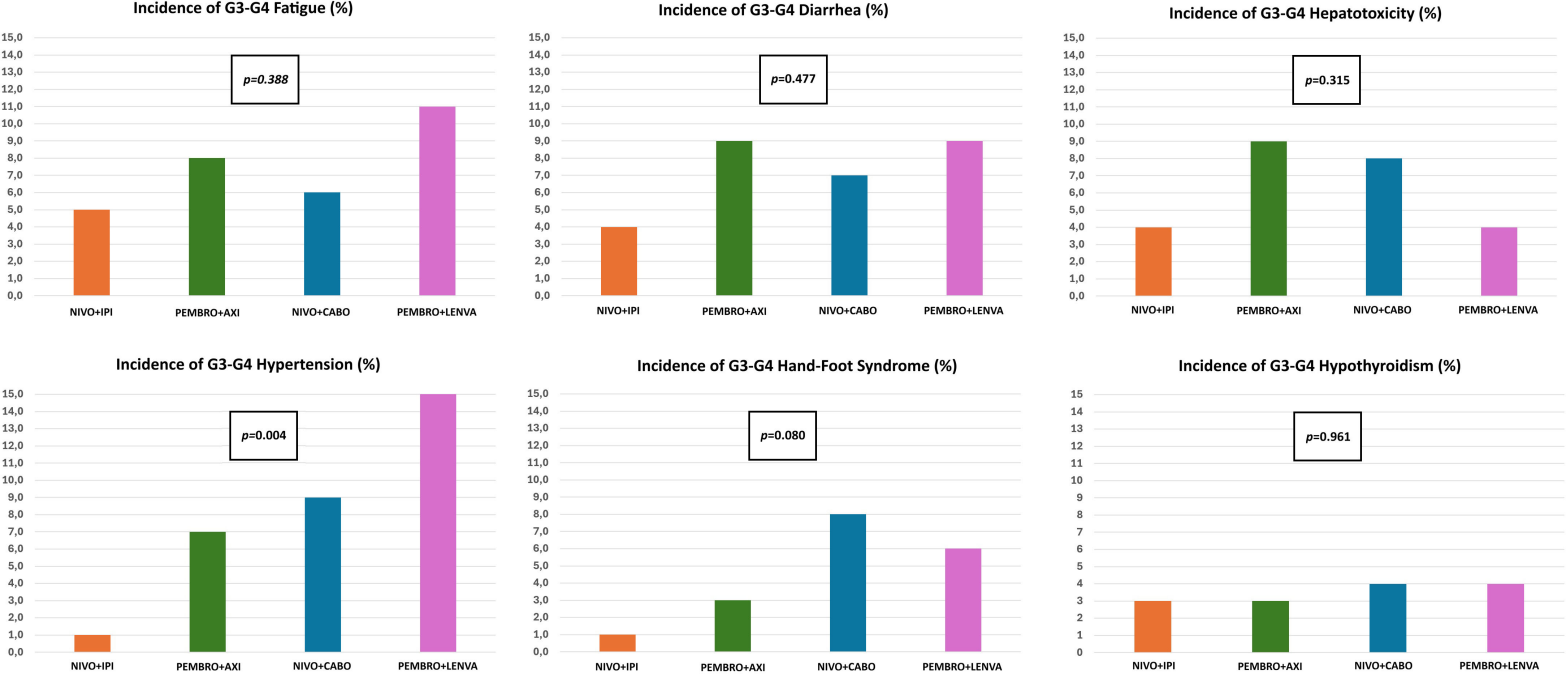
Incidence of grade 3–4 adverse events according to treatment regimen.

We registered 856 G3-G4 AEs in 653 patients, representing 34% of the population. The lowest incidence was observed among patients treated with nivolumab plus ipilimumab (29%), while the highest occurred in those receiving pembrolizumab plus lenvatinib (45%) (**Figure 1**). The most frequent G3-G4 AEs were fatigue (7%) followed by diarrhea (7%), hepatotoxicity (6%) and hypertension (6%). **Figure 2** illustrates the incidence of the most frequent G3-G4 AEs in the study population by treatment type.

Of all G3–4 toxicities compared across the four regimens, only hypertension showed a statistically significant difference in incidence (p=0.004), occurring most frequently in patients receiving pembrolizumab plus lenvatinib (15%), while no statistically significant differences among the combinations were found for all the other G3-G4 AEs. **Table 2** summarizes all treatment-related AEs for each regimen.

**Table 2:** Incidence of G3-G4 Adverse events.

Adverse events	Overall no. (%)	Nivolumab/ ipilimumab (%)	Pembrolizumab/ axitinib (%)	Nivolumab/ cabozantinib (%)	Pembrolizumab/ lenvatinib (%)	*p*
Total patients	1921 (100)	781 (100)	589 (100)	336 (100)	215 (100)	-
G3-G4 Adverse Events	653 (34)	224 (29)	204 (35)	128 (38)	97 (45)	0.127
Fatigue	133 (7)	39 (5)	49 (8)	21 (6)	24 (11)	0.388
Diarrhea	127 (7)	30 (4)	54 (9)	23 (7)	20 (9)	0.477
Hepatotoxicity	113 (6)	28 (4)	51 (9)	26 (8)	8 (4)	0.315
Hypertension	110 (6)	6 (1)	42 (7)	30 (9)	32 (15)	**0.004**
Hand-Foot Syndrome	63 (3)	5 (1)	17 (3)	28 (8)	13 (6)	0.080
Hypothyroidism	62 (3)	25 (3)	15 (3)	13 (4)	9 (4)	0.961
Rash	36 (2)	14 (2)	9 (2)	7 (2)	6 (3)	0.952
Pneumonitis	34 (2)	24 (3)	4 (1)	4 (1)	2 (1)	0.566
Blood creatinine increased	34 (2)	15 (2)	8 (1)	5 (2)	6 (3)	0.796
Arthralgia	23 (1)	12 (2)	4 (1)	4 (1)	3 (1)	0.895
Hematologic toxicity	18 (1)	8 (1)	1 (1)	8 (2)	1 (1)	0.895
Cardiac dysfunctions	15 (1)	5 (1)	6 (1)	2 (1)	2 (1)	1.000
Mucosal inflammation	13 (1)	1 (0)	6 (1)	4 (1)	2 (1)	0.799
Adrenal insufficiency	13 (1)	7 (1)	3 (1)	2 (1)	1 (1)	1.000
Lipase increased	12 (1)	5 (1)	0 (0)	3 (1)	4 (2)	0.568
Nausea	11 (1)	0 (0)	5 (1)	1 (1)	5 (2)	0.568
Neurotoxicity	10 (1)	5 (1)	0 (0)	3 (1)	2 (1)	0.799
Arterial thromboembolic events	10 (1)	3 (1)	3 (1)	3 (1)	1 (1)	1.000
Hypophysitis	9 (<1)	7 (1)	2 (<1)	0 (0)	0 (0)	0.570
Colitis	4 (<1)	4 (<1)	0 (0)	0 (0)	0 (0)	0.391
Venous thromboembolic events	4 (<1)	2 (<1)	2 (<1)	0 (0)	0 (0)	0.570
Hemorrhage	2 (<1)	0 (0)	2 (1)	0 (0)	0 (0)	0.391

Bold values indicate statistical significance (p < 0.05)

### Risk of G3-G4 adverse events and clinical characteristics

The logistic regression analysis demonstrated that both age and sex were significantly associated with the occurrence of G3–4 AEs in the overall study population (**Table 3**). In the multivariable model, increasing age (OR 1.02; 95% CI, 1.01–1.03; p=0.002) and female sex (OR 1.28; 95% CI, 1.01–1.59; p=0.029) were independently associated with a higher risk of severe toxicity. In subgroup analyses, age was significantly correlated with the risk of G3–G4 AEs among patients treated with pembrolizumab plus axitinib (p = 0.015), while in patients receiving nivolumab plus cabozantinib age, IMDC group and liver metastases were associated with a higher risk of G3-G4 AEs (**Table 3**). Conversely, female sex (vs male) was significantly associated with severe AEs in the pembrolizumab plus lenvatinib group (OR 1.92; 95% CI, 1.06–3.49; p=0.032). No statistically significant associations were observed for sex or BMI in the remaining treatment cohorts.

**Table 3:** Logistic regression analysis for grade 3–4 toxicity.

Covariates		Univariable analysis		Multivariable analysis	
		Odds ratio (95%CI)	*p-value*	Odds ratio (95%CI)	*p-value*
Overall Population	Age	1.02 (1.01–1.02)	**<0.001**	1.02 (1.01–1.03)	**0.002**
	Sex (females vs males)	1.25 (1.01–1.54)	**0.039**	1.28 (1.03–1.59)	**0.029**
	Body Mass Index	1.02 (0.99–1.04)	0.056	-	-
	ECOG-PS≥2	0.93 (0.64–1.36)	0.722	-	-
	Prior nephrectomy	1.20 (0.99–1.47)	0.066	-	-
	IMDC intermediate/poor risk group	0.85 (0.73–0.99)	**0.043**	0.86 (0.73–1.01)	0.059
	Lung metastases	1.05 (0.87–1.26)	0.598	-	-
	Liver metastases	0.74 (0.57–0.95)	0.017	-	-
	Brain metastases	1.06 (0.73–1.53)	0.773	-	-
Nivolumab plus Ipilimumab	Age	1.01 (0.99–1.02)	0.227	-	-
	Sex (females vs males)	1.08 (0.76–1.53)	0.662	-	-
	BMI≥25 kg/m^2^	1.01 (0.99–1.06)	0.060	-	-
	ECOG-PS≥2	0.74 (0.40–1.38)	0.342	-	-
	Prior nephrectomy	1.17 (0.84–1.61)	0.356	-	-
	IMDC intermediate/poor risk group	1.22 (0.89–1.67)	0.211	-	-
	Lung metastases	0.86 (0.63–1.24)	0.480	-	-
	Liver metastases	0.63 (0.40–0.98)	**0.040**	-	-
	Brain metastases	0.61 (0.31–1.21)	0.160	-	-
Pembrolizumab plus Axitinib	Age	1.02 (1.01–1.04)	**0.015**	-	-
	Sex (females vs males)	1.15 (0.78–1.71)	0.475	-	-
	BMI≥25 kg/m^2^	1.02 (0.98–1.06)	0.208	-	-
	ECOG-PS≥2	0.80 (0.36–1.78)	0.585	-	-
	Prior nephrectomy	1.16 (0.81–1.65)	0.422	-	-
	IMDC intermediate/poor risk group	0.88 (0.68–1.15)	0.365	-	-
	Lung metastases	1.22 (0.90–1.64)	0.195	-	-
	Liver metastases	1.06 (0.68–1.63)	0.804	-	-
	Brain metastases	1.45 (0.79–2.66)	0.226	-	-
Nivolumab plus Cabozantinib	Age	1.02 (1.01–1.05)	**0.022**	1.02 (1.011.04)	**0.045**
	Sex (females vs males)	1.40 (0.86–2.28)	0.178	-	-
	BMI≥25 kg/m^2^	1.02 (0.97–1.07)	0.414	-	-
	ECOG-PS≥2	1.30 (0.62–2.71)	0.490	-	-
	Prior nephrectomy	1.40 (0.89–2.20)	0.147	-	-
	IMDC intermediate/poor risk group	0.65 (0.47–0.91)	**0.013**	0.69 (0.480.99)	**0.045**
	Lung metastases	1.02 (0.65–2.02)	0.923	-	-
	Liver metastases	0.30 (0.21–0.72)	**0.002**	0.40 (0.210.77)	**0.006**
	Brain metastases	1.69 (0.62–4.64)	0.304	-	-
Pembrolizumab plus lenvatinib	Age	1.02 (0.99–1.04)	0.122	-	-
	Sex (females vs males)	1.92 (1.06–3.49)	**0.032**	-	-
	BMI≥25 kg/m^2^	0.99 (0.95–1.05)	0.899	-	-
	ECOG-PS≥2	1.45 (0.47–4.47)	0.516	-	-
	Prior nephrectomy	1.18 (0.67–2.09)	0.559	-	-
	IMDC intermediate/poor risk group	0.92 (0.63–1.34)	0.665	-	-
	Lung metastases	1.30 (0.74–2.30)	0.358	-	-
	Liver metastases	0.83 (0.44–1.58)	0.570	-	-
	Brain metastases	1.21 (0.41–3.59)	0.727	-	-

Bold values indicate statistical significance (p < 0.05)

### Survival analysis

**Figure 3:**
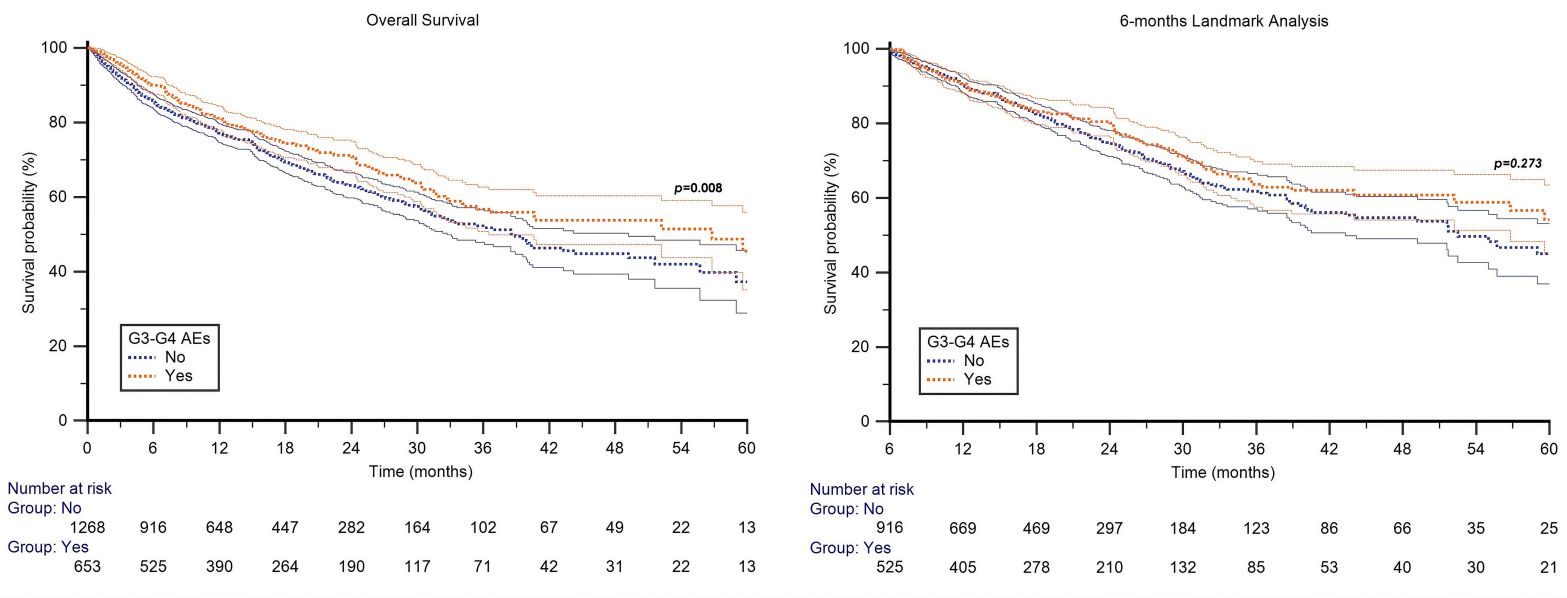
Overall survival and 6-months landmark analysis for patients receiving first-line immune combinations.

The median OS (mOS) was 40.5 months (95%CI, 36.1–55.7) in the overall study population. Patients who experienced G3–4 AEs had a mOS of 56.8 months (95% CI, 36.5–66.5), compared with 38.9 months (95% CI, 32.7–85.3) among those without G3–4 AEs (HR 0.80; 95% CI, 0.67–0.94; p = 0.008; **Figure 3**).

To minimize potential biases related to treatment exposure time, a 6-month landmark analysis for OS was performed. The mOS for the entire cohort was 59.0 months (95% CI, 51.7–168.2). Patients who developed G3–4 AEs had a median OS of 63.5 months (95% CI, 56.8–138.4), compared with 52.5 months (95% CI, 43.3–168.2) in those without G3–4 AEs (HR 0.89; 95% CI, 0.72–1.10; p = 0.273; **Figure 3**).

## Discussion

The selection of first-line therapy for mRCC relies on a careful balance between efficacy and safety based on each patient’s clinical characteristics. Given the lack of direct comparisons among the currently approved immune-based combinations, real-world evidence is essential to better understand safety outcomes and guide individualized treatment decisions in patients outside of clinical trials (19). The present sub-study, derived from the ARON-1 registry, shows real-world safety profiles of the four approved immune-based combinations of mRCC. The demographic and clinical characteristics of our cohort are consistent with those reported in other international real-world series (20, 21). Importantly, this analysis includes patients from 56 centers across 17 different countries, enhancing the external validity of our findings.

In our cohort, the incidence of G3-G4 AEs was 34%, notably lower than that reported in pivotal phase III trials. Among the methodological factors that may contribute to the lower reported rate in retrospective real-world datasets, we should consider differences in follow-up intensity, the retrospective nature of adverse event collection, variability in toxicity attribution and grading across centers, and the potential impact of dose modifications or treatment interruptions in routine clinical practice. Rates of grade ≥3 AEs reached 46% in CheckMate 214, 75% in KEYNOTE-426 and CheckMate 9ER, and 82% in CLEAR (4–7). This difference is likely attributable to the retrospective nature of our study and the inherent limitations of AE reporting and identification in real-world settings, where documentation often relies on the treating physician’s assessment. Nonetheless, these findings underscore the contrast between clinical trials and real-world clinical practice, where patients undergo treatment in a highly controlled scenario with more frequent follow-up and AE assessment (22). In addition, clinical trial participants routinely complete structured questionnaires assessing AEs and HrQoL, whereas in real-world settings there is often a discrepancy between clinician-reported and patient-reported AEs (23, 24).

In our study, the most frequent G3-G4 AEs were fatigue, followed by diarrhea and hepatotoxicity, presenting in 7%, 7% and 6% respectively. Nivolumab plus ipilimumab was associated with the lowest rate of G3-G4 toxicity (29%), whereas pembrolizumab plus lenvatinib showed the highest (45%). These findings align with a meta-analysis comparing first-line phase 3 trials, where pembrolizumab plus lenvatinib demonstrated the highest probability of G3-G4 AEs according to the surface under the cumulative ranking curve (SUCRA) analysis, followed by nivolumab plus cabozantinib. Conversely, nivolumab plus ipilimumab exhibited the lowest SUCRA value, indicating a lower likelihood of high-grade toxicity (25).

The toxicity patterns observed in our study highlight class-specific effects associated with each regimen. For instance, G3-G4 hypertension occurred most frequently among patients receiving lenvatinib-based combination, whereas G3-G4 hand–foot syndrome was more common with cabozantinib-based therapy. This observation is consistent with a meta-analysis of phase III studies assessing IO/TKI combinations in mRCC, where the risk ratio for G3-G4 hypertension was highest with pembrolizumab-based combinations with lenvatinib or axitinib, underscoring the off-target effects of TKIs in the VEGF/VEGFR pathway inhibition (26).

A major clinical challenge for medical oncologists is the overlapping AEs in patients receiving both immunotherapy and TKIs. Hepatotoxicity serves as a representative example. In our study, G3-G4 hepatitis was more frequently reported in patients receiving pembrolizumab plus axitinib, followed by nivolumab plus cabozantinib. The attribution to either immunotherapy or TKI treatment relies on the treating physician and may be biased. While immune-mediated hepatitis results from overactivation of cytotoxic CD8+ T cells, which infiltrate hepatic lobular regions and mediate direct hepatocyte injury, TKI-related hepatic dysfunction is associated with direct hepatic effects from VEGFR inhibition leading to microvascular injury and dysregulation of fibrogenic pathways (27–29).

Therefore, attribution of toxicity may require temporary discontinuation of one or both agents, guided by AE severity, followed by close clinical monitoring to assess reversibility. Given the dose-dependent nature of TKI-related toxicities, improvement after treatment interruption may support a TKI-driven mechanism, whereas persistence or progression of toxicity despite drug suspension may suggest an immune-mediated etiology. In parallel, clinicians must systematically exclude alternative causes, including infectious processes, toxicity from concomitant medications, autoimmune conditions, and disease-related hepatic involvement (30).

The univariate and multivariate analyses showed that older patients and women were at higher risk of AEs. In a previous ARON-1 report including 1, 493 patients, G3-G4 AEs were reported in 32% of men and 35% of women. The frequency of toxicities was consistently higher among female patients, particularly those with a BMI <25 kg/m², where the female-to-male ratio (FMR) reached 1.6. Specific toxicities such as hypothyroidism and diarrhea were also more frequent in women (FMR = 3.0 and 1.5, respectively). Among patients treated with dual immune checkpoint blockade, the overall FMR for severe events was 1.1, increasing to 1.9 in the subgroup with BMI <25 kg/m². Similarly, in those receiving IO/TKI combinations, women experienced higher rates of hypothyroidism (FMR = 2.5), diarrhea (FMR = 2.0), and hypertension (FMR = 1.5), as well as a greater frequency of treatment interruptions (FMR = 1.2). Interestingly, female sex was an independent negative prognostic factor for OS (31). These consistent findings support the hypothesis that sex-related biological differences, including hormonal modulation of immune and vascular pathways, differences in drug metabolism, and body composition may contribute to a heightened susceptibility to both immunotherapy and TKI-related toxicities (32).

The mOS for the entire cohort was 40.5 months (95% CI, 36.1–55.7). Notably, patients who developed G3-G4 AEs demonstrated longer survival as compared to those without severe toxicity (56.8 vs 38.9 months; HR 0.80; 95% CI, 0.67–0.94; p = 0.008). However, the survival difference observed in the unadjusted analysis was not confirmed in the 6-month landmark analysis, suggesting that the association may largely reflect longer treatment exposure among patients who remain on therapy long enough to experience AEs. This interpretation is further supported by prior analyses from our group in the same cohort, which specifically evaluated toxicity-related immunotherapy discontinuation and its clinical implications (33).

The association between treatment-related toxicity and improved outcomes has been widely described in patients receiving immune checkpoint inhibitors, where immune-related AEs often reflect enhanced immune activation against the tumor. Although the evidence is less consistent for TKIs, some data suggest that certain AEs are potential biomarkers of efficacy. For instance, treatment-emergent hypertension has been linked to improved outcomes in patients treated with lenvatinib for thyroid and hepatocellular carcinoma (34, 35). On the other hand, sorafenib-related hand-foot syndrome has been associated with prolonged overall survival in patients with hepatocellular carcinoma (36). These findings support the hypothesis that off-target toxicities may reflect adequate drug exposure or biological sensitivity, translating into improved antitumor activity and survival (37). However, additional data are needed to further characterize this association. Contrarily, the 6-month landmark analysis did not show a statistically significant difference between both groups, this may be in part due to the decreasing incidence of AEs over time (38).

This study has several limitations that should be acknowledged. First, its retrospective design inherently exposes the analysis to potential selection bias, incomplete data capture, and variability in documentation across participating centers. In particular, AEs and clinical outcomes were collected from routine clinical records rather than through prospective, protocol-driven monitoring, which may have resulted in underreporting or heterogeneity in grading and reporting practices. Moreover, we had to initially exclude 20% of patients in the ARON dataset due to the lack of data on G3-G4 AEs, not allowing a complete analysis of the overall population. Additionally, detailed information regarding TKI starting doses was not consistently available across centers, limiting the ability to evaluate the impact of initial dose modifications on both toxicity and clinical outcomes and representing a potential source of residual confounding.

## Conclusion

In this multinational real-world analysis from ARON-1 registry, G3-G4 adverse events occurred in one-third of advanced RCC patients receiving first-line immune-based combinations. The incidence of severe toxicity was lower than in phase III trials, showing differences between controlled trials and routine clinical practice. Toxicity patterns varied across regimens, with pembrolizumab plus lenvatinib showing highest grade adverse events and nivolumab plus ipilimumab the lowest. Older age and female sex increased toxicity risk, highlighting patient-specific factors in treatment decisions. While G3-G4 adverse events correlated with improved survival in unadjusted analyses, this relationship weakened in landmark analyses. These findings demonstrate real-world evidence’s value in supplementing trial data and support individualized toxicity management in clinical practice.

## Data Availability

The raw data supporting the conclusions of this article will be made available by the authors, without undue reservation.
