# Supplementary figures and images for: Real-world incidence of G3-G4 adverse events in patients with advanced renal cell carcinoma receiving immune-combinations (ARON-1)
Source: Front Immunol. 2026 Apr 20;17:1805104. doi: 10.3389/fimmu.2026.1805104 (PMC13136290; doi:10.3389/fimmu.2026.1805104)

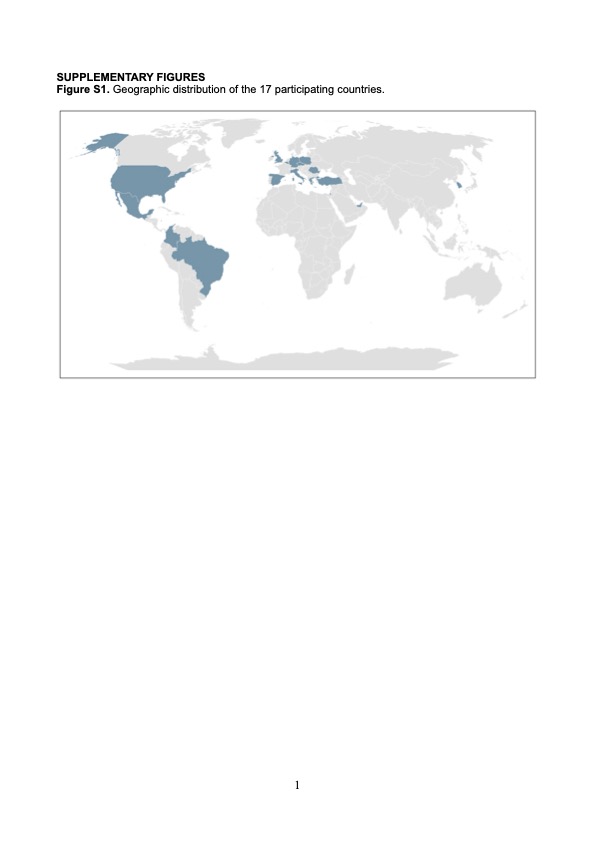

Supplement: Supplementary file 1 [file Image1.jpeg]

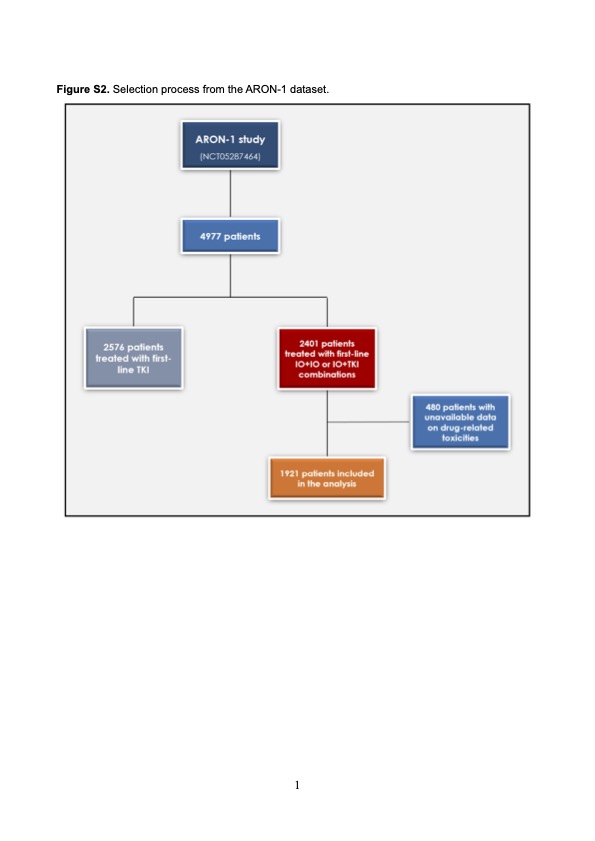

Supplement: Supplementary file 2 [file Image2.jpeg]

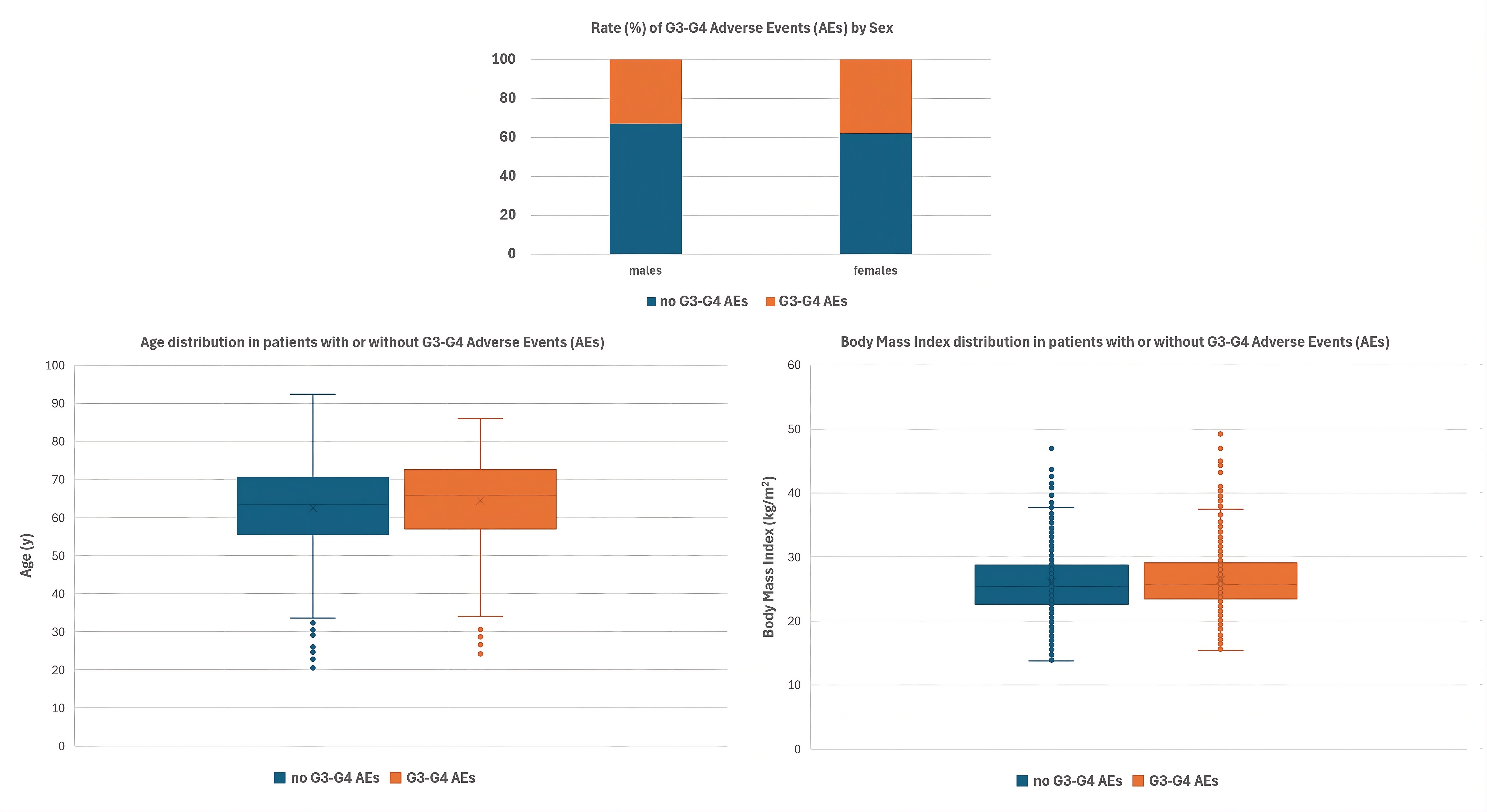

Supplement: Supplementary file 3 [file Image3.jpeg]
